# Supplementary material for: Ageing and Polypharmacy in Mesenchymal Stromal Cells: Metabolic Impact Assessed by Hyperspectral Imaging of Autofluorescence
Source: Int J Mol Sci. 2024 May 27;25(11):5830. doi: 10.3390/ijms25115830 (PMC11171960; doi:10.3390/ijms25115830)
Supplement: Supplementary file 1 [file ijms-25-05830-s001.zip › ijms-3000154-supplementary.pdf]

Supplementary materials

Supplementary Material S1. Python Code used to calculate P-Values between datasets in Python.

```
import numpy as np
import pandas as pd
from scipy import stats

startingColumn = 2

def getPValuesAsStrings(dataFrame1, dataFrame2, startingColumn):
    pValues = []
    for col in range(startingColumn, dataFrame1.columns.size):
        stat, pValue = stats.ttest_ind(dataFrame1.iloc[:, col], dataFrame2.iloc[:, col])
        # corr, pValue = stats.pearsonr(dataFrame1.iloc[:, col], dataFrame2.iloc[:, col])
        pValues.append(pValue)
    return [str(x) for x in pValues]

def listToCsv(inputList, rowNames, columnNames, csvname):
    newDF = pd.DataFrame(inputList, index=rowNames, columns=columnNames)
    newDF.to_csv(csvname)

def main():
    # Import data from csv
    youngC = pd.read_csv("youngC.csv")
    youngP = pd.read_csv("youngP.csv")
    oldC = pd.read_csv("oldC.csv")
    oldP = pd.read_csv("oldP.csv")

    rowNames = youngC.columns[startingColumn:]
    columnNames = ['P-Values']

    pValuesYCvsOCList = getPValuesAsStrings(youngC, oldC, startingColumn)
    listToCsv(pValuesYCvsOCList, rowNames, columnNames, 'youngC-vs-oldC.csv')

    pValuesYCvsYPList = getPValuesAsStrings(youngC, youngP, startingColumn)
    listToCsv(pValuesYCvsYPList, rowNames, columnNames, 'youngC-vs-youngP.csv')

    pValuesYCvsOPList = getPValuesAsStrings(youngC, oldP, startingColumn)
    listToCsv(pValuesYCvsOPList, rowNames, columnNames, 'youngC-vs-oldP.csv')

    pValuesOCvsYPList = getPValuesAsStrings(oldC, youngP, startingColumn)
    listToCsv(pValuesOCvsYPList, rowNames, columnNames, 'oldC-vs-youngP.csv')

    pValuesOCvsOPList = getPValuesAsStrings(oldC, oldP, startingColumn)
    listToCsv(pValuesOCvsOPList, rowNames, columnNames, 'oldC-vs-oldP.csv')

    pValuesYPvsOPList = getPValuesAsStrings(youngP, oldP, startingColumn)
    listToCsv(pValuesYPvsOPList, rowNames, columnNames, 'youngP-vs-oldP.csv')

if __name__ == "__main__":
    main()
```

Supplementary Material S2. MATLAB Script used to calculate ORR.

```
YC=table2array(stats);
OC=table2array(statsS1);
YP=table2array(statsS2);
OP=table2array(statsS3);

%Choose flavin and nadh channels
GROUP_NAME_flav = YC(:,[8,9,26]);
GROUP_NAME_nadh = YC(:,[1,10,11,12]);

%multiplication of NADH or flavins
[origrowsx, origcolsx] = size(GROUP_NAME_nadh); %nadh here
[origrowsy, origcolsy] = size(GROUP_NAME_flav); %flavins here
colsy_sq = origcolsx * origcolsy;

%make matrix with all columns repeated
GROUP_NAME_nadh_allcol_redox = zeros(origrowsx, colsy_sq);
for row = 1: origrowsx
    for col = 1:origcolsy
        filler = (col - 1) * origcolsx;
        for colfil = 1:origcolsx
            GROUP_NAME_nadh_allcol_redox(row, filler + colfil) = GROUP_NAME_nadh(row,colfil);
        end
    end
end

%make matrix with each column repeated
GROUP_NAME_flav_eachcol_redox = zeros(origrowsx, colsy_sq);
for row = 1: origrowsy
    for col = 1:origcolsy
        filler = (col - 1) * origcolsx;
        for colfil = 1:origcolsx
            GROUP_NAME_flav_eachcol_redox(row, filler + colfil) = GROUP_NAME_flav(row,col);
        end
    end
end

%Redox Ratio = NADH/Flavins
GROUP_NAME_redox = zeros(origrowsx+1, colsy_sq);
for row = 1 : origrowsx
  for col = 1 : colsy_sq
      GROUP_NAME_redox(row+1, col) = GROUP_NAME_nadh_allcol_redox(row, col) / GROUP_NAME_flav_eachcol_redox(row, col);
  end
end


writematrix(GROUP_NAME_redox,'YC_YCvOP_redox.xlsx')
writematrix(GROUP_NAME_redox,'YC_YCvOP_redox.csv')
```

Supplementary Material S3. MATLAB Script used to calculate Secondary Channels.

```
YC=table2array(stats);
OC=table2array(statsS1);
YP=table2array(statsS2);
OP=table2array(statsS3);

%YC vs OP ratios
GROUP_NAME_flav = OC(:,[8,9,25,31]);
GROUP_NAME_nadh = OC(:,[1]);

%multiplication of NADH or flavins
[origrowsx, origcolsx] = size(GROUP_NAME_flav);
[origrowsy, origcolsy] = size(GROUP_NAME_flav);
colsy_sq = origcolsy * origcolsy;
%make matrix with all columns repeated
GROUP_NAME_flav_allcol = zeros(origrowsx, colsy_sq);
for row = 1: origrowsx
    for col = 1:origcolsy
        filler = (col - 1) * origcolsy;
        for colfil = 1:origcolsy
            GROUP_NAME_flav_allcol(row, filler + colfil) = GROUP_NAME_flav(row,colfil);
        end
    end
end

%make matrix with each column repeated
GROUP_NAME_flav_eachcol = zeros(origrowsx, colsy_sq);
for row = 1: origrowsx
    for col = 1:origcolsy
        filler = (col - 1) * origcolsy;
        for colfil = 1:origcolsy
            GROUP_NAME_flav_eachcol(row, filler + colfil) = GROUP_NAME_flav(row,col);
        end
    end
end

GROUP_NAME_flav_prod = zeros(origrowsx, colsy_sq);
for row = 1 : origrowsx
  for col = 1 : colsy_sq
      GROUP_NAME_flav_prod(row, col) = GROUP_NAME_flav_allcol(row, col) * GROUP_NAME_flav_eachcol(row, col);
  end
end

writematrix(GROUP_NAME_flav_prod,'OC_OCvYP_flav_prod.xlsx')
%writematrix(GROUP_NAME_flav_prod,'YC_YCvOC_flav_prod.csv')

```

```

%multiplication of NADH or flavins
[origrowsx, origcolsx] = size(GROUP_NAME_nadh);
[origrowsy, origcolsy] = size(GROUP_NAME_nadh);
colsy_sq = origcolsy * origcolsy;
%make matrix with all columns repeated
GROUP_NAME_nadh_allcol = zeros(origrowsx, colsy_sq);
for row = 1: origrowsx
    for col = 1:origcolsy
        filler = (col - 1) * origcolsy;
        for colfil = 1:origcolsy
            GROUP_NAME_nadh_allcol(row, filler + colfil) = GROUP_NAME_nadh(row,colfil);
        end
    end
end
%make matrix with each column repeated
GROUP_NAME_nadh_eachcol = zeros(origrowsx, colsy_sq);
for row = 1: origrowsx
    for col = 1:origcolsy
        filler = (col - 1) * origcolsy;
        for colfil = 1:origcolsy
            GROUP_NAME_nadh_eachcol(row, filler + colfil) = GROUP_NAME_nadh(row,col);
        end
    end
end

GROUP_NAME_nadh_prod = zeros(origrowsx, colsy_sq);
for row = 1 : origrowsx
  for col = 1 : colsy_sq
      GROUP_NAME_nadh_prod(row, col) = GROUP_NAME_nadh_allcol(row, col) * GROUP_NAME_nadh_eachcol(row, col);
  end
end

writematrix(GROUP_NAME_nadh_prod,'OC_OCvYP_nadh_prod.xlsx')
%writematrix(GROUP_NAME_nadh_prod,'OC_YCvOC_nadh_prod.csv')

```

Supplementary Material S4. Non significant findings.

	P-Values						p < 0.05?	Fluorophore		p < 0.001?
Channel_1	0.63234		Channel_31	6.78E-07		Channel_1	no	NADH/Elastin	Channel_1	no
Channel_2	0.661354		Channel_19	4.29E-06		Channel_2	no	NADH	Channel_2	no
Channel_3	0.671633		Channel_30	3.34E-05		Channel_3	no	Flavin/Lipo-Pig	Channel_3	no
Channel_4	0.014078		Channel_25	0.000522		Channel_4	0.014078	Flavin	Channel_4	no
Channel_5	0.029465		Channel_17	0.000668		Channel_5	0.029465	Flavin	Channel_5	no
Channel_6	0.001686		Channel_11	0.000808		Channel_6	0.001686	Flavin	Channel_6	no
Channel_7	0.015851		Channel_6	0.001686		Channel_7	0.015851	Flavin	Channel_7	no
Channel_8	0.941232		Channel_28	0.001958		Channel_8	no	Porphyrin	Channel_8	no
Channel_9	0.336192		Channel_22	0.003292		Channel_9	no	Porphyrin	Channel_9	no
Channel_10	0.068137		Channel_18	0.004832		Channel_10	no	NADH/Elastin	Channel_10	no
Channel_11	0.000808		Channel_29	0.013391		Channel_11	0.000808	NADH/Elastin	Channel_11	0.000808
Channel_12	0.061872		Channel_4	0.014078		Channel_12	no	NADH/Elastin	Channel_12	no
Channel_13	0.234611		Channel_24	0.015346		Channel_13	no	NADH/Elastin	Channel_13	no
Channel_14	0.034579		Channel_7	0.015851		Channel_14	0.034579	Flavin	Channel_14	no
Channel_15	0.034652		Channel_21	0.01892		Channel_15	0.034652	Flavin	Channel_15	no
Channel_16	0.039073		Channel_20	0.021949		Channel_16	0.039073	Flavin	Channel_16	no
Channel_17	0.000668		Channel_5	0.029465		Channel_17	0.000668	Flavin	Channel_17	0.000668
Channel_18	0.004832		Channel_14	0.034579		Channel_18	0.004832	Flavin	Channel_18	no
Channel_19	4.29E-06		Channel_15	0.034652		Channel_19	4.29E-06	Flavin/Lipo-Pig	Channel_19	4.29E-06
Channel_20	0.021949		Channel_16	0.039073		Channel_20	0.021949	Flavin/Lipo-Pig	Channel_20	no
Channel_21	0.01892		Channel_27	0.057205		Channel_21	0.01892	Flavin	Channel_21	no
Channel_22	0.003292		Channel_12	0.061872		Channel_22	0.003292	Flavin	Channel_22	no
Channel_23	0.145933		Channel_10	0.068137		Channel_23	no	Flavin	Channel_23	no
Channel_24	0.015346		Channel_23	0.145933		Channel_24	0.015346	Flavin	Channel_24	no
Channel_25	0.000522		Channel_13	0.234611		Channel_25	0.000522	Flavin	Channel_25	0.000522
Channel_26	0.30442		Channel_26	0.30442		Channel_26	no	Porphyrin	Channel_26	no
Channel_27	0.057205		Channel_9	0.336192		Channel_27	no	Porphyrin	Channel_27	no
Channel_28	0.001958		Channel_1	0.63234		Channel_28	0.001958	Porphyrin	Channel_28	no
Channel_29	0.013391		Channel_2	0.661354		Channel_29	0.013391	Porphyrin	Channel_29	no
Channel_30	3.34E-05		Channel_3	0.671633		Channel_30	3.34E-05	Flavin/Porphyrin	Channel_30	3.34E-05
Channel_31	6.78E-07		Channel_8	0.941232		Channel_31	6.78E-07	Flavin/Porphyrin	Channel_31	6.78E-07
Channel_32										

Significance of all hyperspectral channels in the comparison of Young Control Cells and Old Control Cells.

	P-Values						p < 0.05?	Fluorophore		p < 0.001?
Channel_1	0.60631		Channel_26	0.058858		Channel_1	no	NADH/Elastin	Channel_1	no
Channel_2	0.932366		Channel_28	0.179719		Channel_2	no	NADH	Channel_2	no
Channel_3	0.994423		Channel_10	0.232852		Channel_3	no	Flavin/Lipo-Pig	Channel_3	no
Channel_4	0.489158		Channel_18	0.256073		Channel_4	no	Flavin	Channel_4	no
Channel_5	0.475096		Channel_27	0.318784		Channel_5	no	Flavin	Channel_5	no
Channel_6	0.882181		Channel_16	0.338534		Channel_6	no	Flavin	Channel_6	no
Channel_7	0.70101		Channel_31	0.369329		Channel_7	no	Flavin	Channel_7	no
Channel_8	0.641103		Channel_17	0.389478		Channel_8	no	Porphyrin	Channel_8	no
Channel_9	0.648753		Channel_25	0.410264		Channel_9	no	Porphyrin	Channel_9	no
Channel_10	0.232852		Channel_14	0.413964		Channel_10	no	NADH/Elastin	Channel_10	no
Channel_11	0.586369		Channel_5	0.475096		Channel_11	no	NADH/Elastin	Channel_11	no
Channel_12	0.574625		Channel_29	0.47854		Channel_12	no	NADH/Elastin	Channel_12	no
Channel_13	0.902114		Channel_4	0.489158		Channel_13	no	NADH/Elastin	Channel_13	no
Channel_14	0.413964		Channel_12	0.574625		Channel_14	no	Flavin	Channel_14	no
Channel_15	0.708751		Channel_11	0.586369		Channel_15	no	Flavin	Channel_15	no
Channel_16	0.338534		Channel_1	0.60631		Channel_16	no	Flavin	Channel_16	no
Channel_17	0.389478		Channel_8	0.641103		Channel_17	no	Flavin	Channel_17	no
Channel_18	0.256073		Channel_9	0.648753		Channel_18	no	Flavin	Channel_18	no
Channel_19	0.762619		Channel_30	0.652712		Channel_19	no	Flavin/Lipo-Pig	Channel_19	no
Channel_20	0.765669		Channel_7	0.70101		Channel_20	no	Flavin/Lipo-Pig	Channel_20	no
Channel_21	0.779717		Channel_15	0.708751		Channel_21	no	Flavin	Channel_21	no
Channel_22	0.753142		Channel_23	0.739877		Channel_22	no	Flavin	Channel_22	no
Channel_23	0.739877		Channel_22	0.753142		Channel_23	no	Flavin	Channel_23	no
Channel_24	0.800273		Channel_19	0.762619		Channel_24	no	Flavin	Channel_24	no
Channel_25	0.410264		Channel_20	0.765669		Channel_25	no	Flavin	Channel_25	no
Channel_26	0.058858		Channel_21	0.779717		Channel_26	no	Porphyrin	Channel_26	no
Channel_27	0.318784		Channel_24	0.800273		Channel_27	no	Porphyrin	Channel_27	no
Channel_28	0.179719		Channel_6	0.882181		Channel_28	no	Porphyrin	Channel_28	no
Channel_29	0.47854		Channel_13	0.902114		Channel_29	no	Porphyrin	Channel_29	no
Channel_30	0.652712		Channel_2	0.932366		Channel_30	no	Flavin/Porphyrin	Channel_30	no
Channel_31	0.369329		Channel_3	0.994423		Channel_31	no	Flavin/Porphyrin	Channel_31	no
Channel_32										

Significance of all hyperspectral channels in the comparison of Young Polypharmacy Treated Cells and Old Polypharmacy Treated Cells.

	P-Values						p < 0.05?	Fluorophore		p < 0.001?
Channel_1	0.001356		Channel_9	8.77E-06		Channel_1	0.0013563	NADH/Elastin	Channel_1	no
Channel_2	0.021616		Channel_8	8.77E-05		Channel_2	0.0216156	NADH	Channel_2	no
Channel_3	0.041011		Channel_10	0.000525		Channel_3	0.0410113	Flavin/Lipo-Pig	Channel_3	no
Channel_4	0.24019		Channel_11	0.001079		Channel_4	no	Flavin	Channel_4	no
Channel_5	0.775903		Channel_1	0.001356		Channel_5	no	Flavin	Channel_5	no
Channel_6	0.727891		Channel_12	0.010911		Channel_6	no	Flavin	Channel_6	no
Channel_7	0.830156		Channel_13	0.011741		Channel_7	no	Flavin	Channel_7	no
Channel_8	8.77E-05		Channel_14	0.018332		Channel_8	8.773E-05	Porphyrin	Channel_8	8.77E-05
Channel_9	8.77E-06		Channel_2	0.021616		Channel_9	8.765E-06	Porphyrin	Channel_9	8.77E-06
Channel_10	0.000525		Channel_15	0.035833		Channel_10	0.0005246	NADH/Elastin	Channel_10	0.000525
Channel_11	0.001079		Channel_3	0.041011		Channel_11	0.0010788	NADH/Elastin	Channel_11	no
Channel_12	0.010911		Channel_26	0.097524		Channel_12	0.0109107	NADH/Elastin	Channel_12	no
Channel_13	0.011741		Channel_17	0.173876		Channel_13	0.0117411	NADH/Elastin	Channel_13	no
Channel_14	0.018332		Channel_23	0.233363		Channel_14	0.018332	Flavin	Channel_14	no
Channel_15	0.035833		Channel_4	0.24019		Channel_15	0.0358328	Flavin	Channel_15	no
Channel_16	0.354909		Channel_30	0.353501		Channel_16	no	Flavin	Channel_16	no
Channel_17	0.173876		Channel_16	0.354909		Channel_17	no	Flavin	Channel_17	no
Channel_18	0.491983		Channel_24	0.3848		Channel_18	no	Flavin	Channel_18	no
Channel_19	0.386453		Channel_19	0.386453		Channel_19	no	Flavin/Lipo-Pig	Channel_19	no
Channel_20	0.557853		Channel_25	0.433372		Channel_20	no	Flavin/Lipo-Pig	Channel_20	no
Channel_21	0.718764		Channel_18	0.491983		Channel_21	no	Flavin	Channel_21	no
Channel_22	0.812189		Channel_20	0.557853		Channel_22	no	Flavin	Channel_22	no
Channel_23	0.233363		Channel_21	0.718764		Channel_23	no	Flavin	Channel_23	no
Channel_24	0.3848		Channel_31	0.722327		Channel_24	no	Flavin	Channel_24	no
Channel_25	0.433372		Channel_6	0.727891		Channel_25	no	Flavin	Channel_25	no
Channel_26	0.097524		Channel_5	0.775903		Channel_26	no	Porphyrin	Channel_26	no
Channel_27	0.867389		Channel_22	0.812189		Channel_27	no	Porphyrin	Channel_27	no
Channel_28	0.955506		Channel_7	0.830156		Channel_28	no	Porphyrin	Channel_28	no
Channel_29	0.868213		Channel_27	0.867389		Channel_29	no	Porphyrin	Channel_29	no
Channel_30	0.353501		Channel_29	0.868213		Channel_30	no	Flavin/Porphyrin	Channel_30	no
Channel_31	0.722327		Channel_28	0.955506		Channel_31	no	Flavin/Porphyrin	Channel_31	no
Channel_32										

Significance of all hyperspectral channels in the comparison of Young Control Cells and Young Polypharmacy Treated Cells.

	P-Values						p < 0.05?	Fluorophore		p < 0.001?
Channel_1	0.024463		Channel_28	2.61E-05		Channel_1	0.024463	NADH/Elastin	Channel_1	no
Channel_2	0.051587		Channel_26	7.89E-05		Channel_2	no	NADH	Channel_2	no
Channel_3	0.075219		Channel_8	0.000422		Channel_3	no	Flavin/Lipo-Pig	Channel_3	no
Channel_4	0.696175		Channel_9	0.001072		Channel_4	no	Flavin	Channel_4	no
Channel_5	0.362344		Channel_19	0.001328		Channel_5	no	Flavin	Channel_5	no
Channel_6	0.010458		Channel_27	0.001926		Channel_6	0.010458	Flavin	Channel_6	no
Channel_7	0.005873		Channel_29	0.003921		Channel_7	0.005873	Flavin	Channel_7	no
Channel_8	0.000422		Channel_31	0.004531		Channel_8	0.000422	Porphyrin	Channel_8	0.000422
Channel_9	0.001072		Channel_25	0.00472		Channel_9	0.001072	Porphyrin	Channel_9	no
Channel_10	0.030899		Channel_7	0.005873		Channel_10	0.030899	NADH/Elastin	Channel_10	no
Channel_11	0.59406		Channel_6	0.010458		Channel_11	no	NADH/Elastin	Channel_11	no
Channel_12	0.25		Channel_24	0.014853		Channel_12	no	NADH/Elastin	Channel_12	no
Channel_13	0.11444		Channel_1	0.024463		Channel_13	no	NADH/Elastin	Channel_13	no
Channel_14	0.309744		Channel_30	0.027273		Channel_14	no	Flavin	Channel_14	no
Channel_15	0.648444		Channel_10	0.030899		Channel_15	no	Flavin	Channel_15	no
Channel_16	0.816611		Channel_22	0.042669		Channel_16	no	Flavin	Channel_16	no
Channel_17	0.212457		Channel_2	0.051587		Channel_17	no	Flavin	Channel_17	no
Channel_18	0.295775		Channel_23	0.052018		Channel_18	no	Flavin	Channel_18	no
Channel_19	0.001328		Channel_3	0.075219		Channel_19	0.001328	Flavin/Lipo-Pig	Channel_19	no
Channel_20	0.210597		Channel_13	0.11444		Channel_20	no	Flavin/Lipo-Pig	Channel_20	no
Channel_21	0.134688		Channel_21	0.134688		Channel_21	no	Flavin	Channel_21	no
Channel_22	0.042669		Channel_20	0.210597		Channel_22	0.042669	Flavin	Channel_22	no
Channel_23	0.052018		Channel_17	0.212457		Channel_23	no	Flavin	Channel_23	no
Channel_24	0.014853		Channel_12	0.25		Channel_24	0.014853	Flavin	Channel_24	no
Channel_25	0.00472		Channel_18	0.295775		Channel_25	0.00472	Flavin	Channel_25	no
Channel_26	7.89E-05		Channel_14	0.309744		Channel_26	7.89E-05	Porphyrin	Channel_26	7.89E-05
Channel_27	0.001926		Channel_5	0.362344		Channel_27	0.001926	Porphyrin	Channel_27	no
Channel_28	2.61E-05		Channel_11	0.59406		Channel_28	2.61E-05	Porphyrin	Channel_28	2.61E-05
Channel_29	0.003921		Channel_15	0.648444		Channel_29	0.003921	Porphyrin	Channel_29	no
Channel_30	0.027273		Channel_4	0.696175		Channel_30	0.027273	Flavin/Porphyrin	Channel_30	no
Channel_31	0.004531		Channel_16	0.816611		Channel_31	0.004531	Flavin/Porphyrin	Channel_31	no
Channel_32										

Significance of all hyperspectral channels in the comparison of Old Control Cells and Old Polypharmacy Treated Cells.

	P-Values						p < 0.05?	Fluorophore		p < 0.001?
Channel_1	0.004317		Channel_31	4.93E-05		Channel_1	0.004317	NADH/Elastin	Channel_1	no
Channel_2	0.03691		Channel_8	5.23E-05		Channel_2	0.03691	NADH	Channel_2	no
Channel_3	0.068384		Channel_25	7.09E-05		Channel_3	no	Flavin/Lipo-Pig	Channel_3	no
Channel_4	0.263289		Channel_9	0.000121		Channel_4	no	Flavin	Channel_4	no
Channel_5	0.094674		Channel_19	0.002115		Channel_5	no	Flavin	Channel_5	no
Channel_6	0.01606		Channel_24	0.002155		Channel_6	0.01606	Flavin	Channel_6	no
Channel_7	0.022676		Channel_30	0.00336		Channel_7	0.022676	Flavin	Channel_7	no
Channel_8	5.23E-05		Channel_28	0.004167		Channel_8	5.23E-05	Porphyrin	Channel_8	5.23E-05
Channel_9	0.000121		Channel_1	0.004317		Channel_9	0.000121	Porphyrin	Channel_9	0.000121
Channel_10	0.206837		Channel_22	0.009901		Channel_10	no	NADH/Elastin	Channel_10	no
Channel_11	0.973236		Channel_23	0.011099		Channel_11	no	NADH/Elastin	Channel_11	no
Channel_12	0.502862		Channel_6	0.01606		Channel_12	no	NADH/Elastin	Channel_12	no
Channel_13	0.123057		Channel_7	0.022676		Channel_13	no	NADH/Elastin	Channel_13	no
Channel_14	0.779997		Channel_29	0.028509		Channel_14	no	Flavin	Channel_14	no
Channel_15	0.913629		Channel_18	0.030151		Channel_15	no	Flavin	Channel_15	no
Channel_16	0.231126		Channel_26	0.035385		Channel_16	no	Flavin	Channel_16	no
Channel_17	0.035743		Channel_17	0.035743		Channel_17	0.035743	Flavin	Channel_17	no
Channel_18	0.030151		Channel_2	0.03691		Channel_18	0.030151	Flavin	Channel_18	no
Channel_19	0.002115		Channel_27	0.048553		Channel_19	0.002115	Flavin/Lipo-Pig	Channel_19	no
Channel_20	0.103328		Channel_21	0.058677		Channel_20	no	Flavin/Lipo-Pig	Channel_20	no
Channel_21	0.058677		Channel_3	0.068384		Channel_21	no	Flavin	Channel_21	no
Channel_22	0.009901		Channel_5	0.094674		Channel_22	0.009901	Flavin	Channel_22	no
Channel_23	0.011099		Channel_20	0.103328		Channel_23	0.011099	Flavin	Channel_23	no
Channel_24	0.002155		Channel_13	0.123057		Channel_24	0.002155	Flavin	Channel_24	no
Channel_25	7.09E-05		Channel_10	0.206837		Channel_25	7.09E-05	Flavin	Channel_25	7.09E-05
Channel_26	0.035385		Channel_16	0.231126		Channel_26	0.035385	Porphyrin	Channel_26	no
Channel_27	0.048553		Channel_4	0.263289		Channel_27	0.048553	Porphyrin	Channel_27	no
Channel_28	0.004167		Channel_12	0.502862		Channel_28	0.004167	Porphyrin	Channel_28	no
Channel_29	0.028509		Channel_14	0.779997		Channel_29	0.028509	Porphyrin	Channel_29	no
Channel_30	0.00336		Channel_15	0.913629		Channel_30	0.00336	Flavin/Porphyrin	Channel_30	no
Channel_31	4.93E-05		Channel_11	0.973236		Channel_31	4.93E-05	Flavin/Porphyrin	Channel_31	4.93E-05
Channel_32										

Significance of all hyperspectral channels in the comparison of Old Control Cells and Young Polypharmacy Treated Cells.

	P-Values						p < 0.05?	Fluorophore		p < 0.001?
Channel_1	0.009202		Channel_10	1.31E-05		Channel_1	0.009202	NADH/Elastin	Channel_1	no
Channel_2	0.031425		Channel_26	7.45E-05		Channel_2	0.031425	NADH	Channel_2	no
Channel_3	0.047022		Channel_9	0.000102		Channel_3	0.047022	Flavin/Lipo-Pig	Channel_3	no
Channel_4	0.060358		Channel_8	0.000662		Channel_4	no	Flavin	Channel_4	no
Channel_5	0.283671		Channel_14	0.002372		Channel_5	no	Flavin	Channel_5	no
Channel_6	0.858973		Channel_12	0.003191		Channel_6	no	Flavin	Channel_6	no
Channel_7	0.505535		Channel_11	0.007239		Channel_7	no	Flavin	Channel_7	no
Channel_8	0.000662		Channel_1	0.009202		Channel_8	0.000662	Porphyrin	Channel_8	0.000662
Channel_9	0.000102		Channel_13	0.012488		Channel_9	0.000102	Porphyrin	Channel_9	0.000102
Channel_10	1.31E-05		Channel_15	0.020712		Channel_10	1.31E-05	NADH/Elastin	Channel_10	1.31E-05
Channel_11	0.007239		Channel_2	0.031425		Channel_11	0.007239	NADH/Elastin	Channel_11	no
Channel_12	0.003191		Channel_17	0.038755		Channel_12	0.003191	NADH/Elastin	Channel_12	no
Channel_13	0.012488		Channel_3	0.047022		Channel_13	0.012488	NADH/Elastin	Channel_13	no
Channel_14	0.002372		Channel_4	0.060358		Channel_14	0.002372	Flavin	Channel_14	no
Channel_15	0.020712		Channel_18	0.092643		Channel_15	0.020712	Flavin	Channel_15	no
Channel_16	0.094065		Channel_16	0.094065		Channel_16	no	Flavin	Channel_16	no
Channel_17	0.038755		Channel_28	0.173276		Channel_17	0.038755	Flavin	Channel_17	no
Channel_18	0.092643		Channel_31	0.177808		Channel_18	no	Flavin	Channel_18	no
Channel_19	0.653889		Channel_30	0.182381		Channel_19	no	Flavin/Lipo-Pig	Channel_19	no
Channel_20	0.392033		Channel_27	0.225359		Channel_20	no	Flavin/Lipo-Pig	Channel_20	no
Channel_21	0.532072		Channel_5	0.283671		Channel_21	no	Flavin	Channel_21	no
Channel_22	0.587339		Channel_20	0.392033		Channel_22	no	Flavin	Channel_22	no
Channel_23	0.469439		Channel_23	0.469439		Channel_23	no	Flavin	Channel_23	no
Channel_24	0.624123		Channel_7	0.505535		Channel_24	no	Flavin	Channel_24	no
Channel_25	0.873759		Channel_21	0.532072		Channel_25	no	Flavin	Channel_25	no
Channel_26	7.45E-05		Channel_29	0.572607		Channel_26	7.45E-05	Porphyrin	Channel_26	7.45E-05
Channel_27	0.225359		Channel_22	0.587339		Channel_27	no	Porphyrin	Channel_27	no
Channel_28	0.173276		Channel_24	0.624123		Channel_28	no	Porphyrin	Channel_28	no
Channel_29	0.572607		Channel_19	0.653889		Channel_29	no	Porphyrin	Channel_29	no
Channel_30	0.182381		Channel_6	0.858973		Channel_30	no	Flavin/Porphyrin	Channel_30	no
Channel_31	0.177808		Channel_25	0.873759		Channel_31	no	Flavin/Porphyrin	Channel_31	no
Channel_32										

Significance of all hyperspectral channels in the comparison of Young Control Cells and Old Polypharmacy Treated Cells.

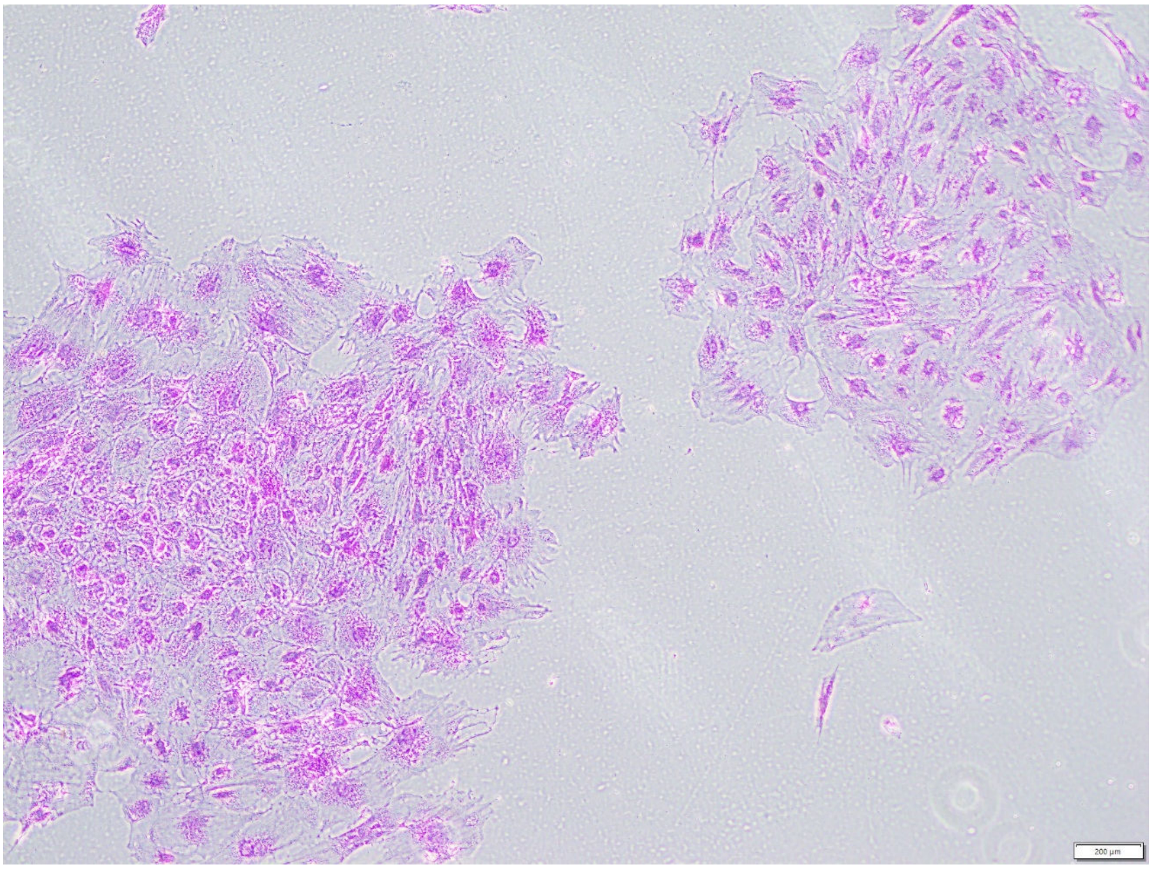

Supplementary figure S1: Colony forming units. Representative image of two MSC colony forming unit colonies stained with crystal violet from old, polypharmacy exposed mice

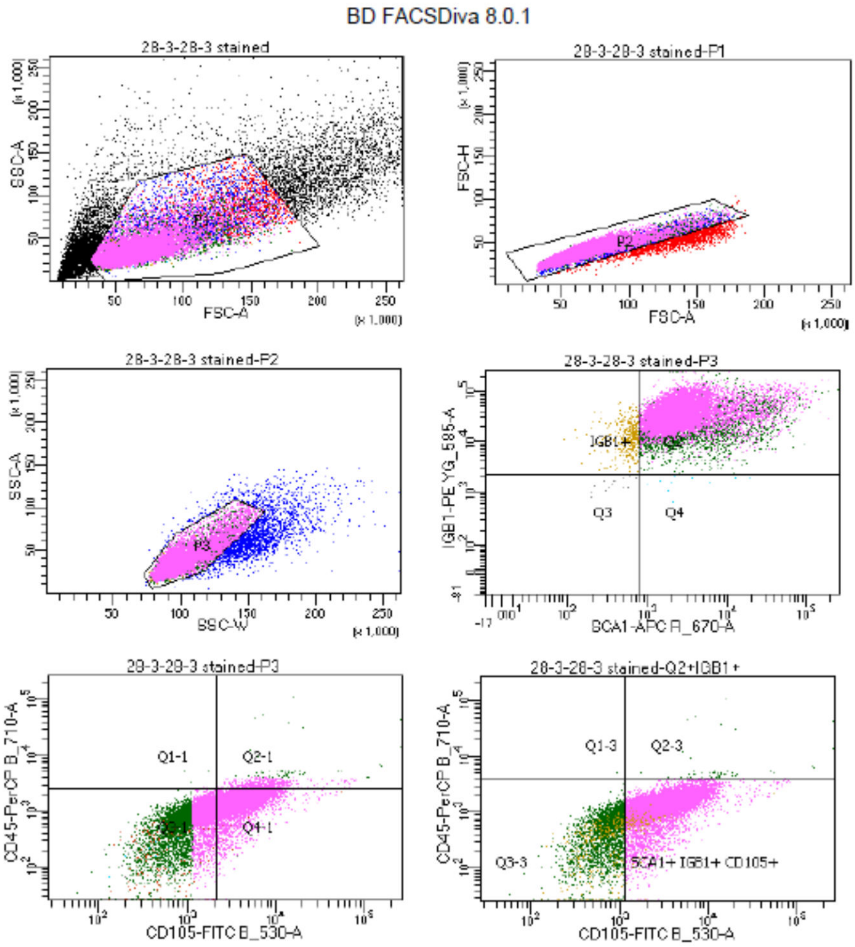

Tube: 28-3 stained

Population	#Events	%Parent	%Total
All Events	30,000	####	100.0
P1	21,469	71.6	71.6
P2	19,572	91.2	65.2
P3	17,331	88.5	57.8
IGB1+	432	2.5	1.4
Q2	16,875	97.4	56.2
Q1-3	1	0.0	0.0
Q2-3	60	0.4	0.2
Q3-3	2,980	17.7	9.9
SCA1+ IGB1+ CD105+	13,834	82.0	46.1
Q3	14	0.1	0.0
Q4	10	0.1	0.0
Q1-1	5	0.0	0.0
Q2-1	650	3.8	2.2
Q3-1	7,455	43.0	24.8
Q4-1	9,221	53.2	30.7

Supplementary Figure S2. Cells surface markers. Representative MSC cell surface marker staining from MSC line derived from a young, polypharmacy exposed mouse.

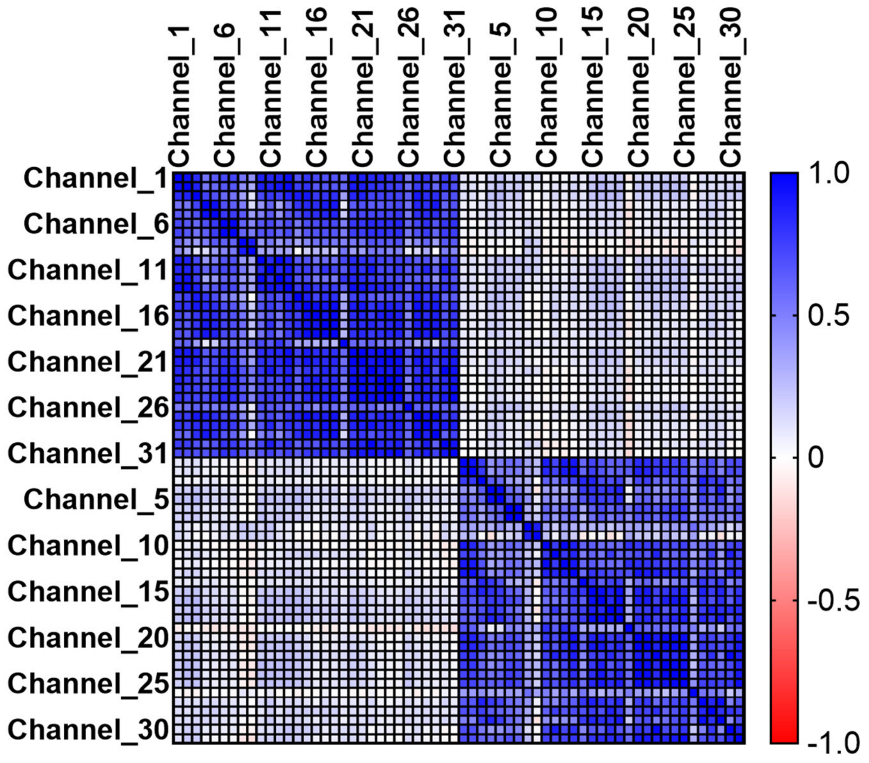

Supplementary Figure S3. Correlation Matrix between 2 groups of cells, the top right quadrant is used to gather the relevant Pearson Correlation Coefficients.

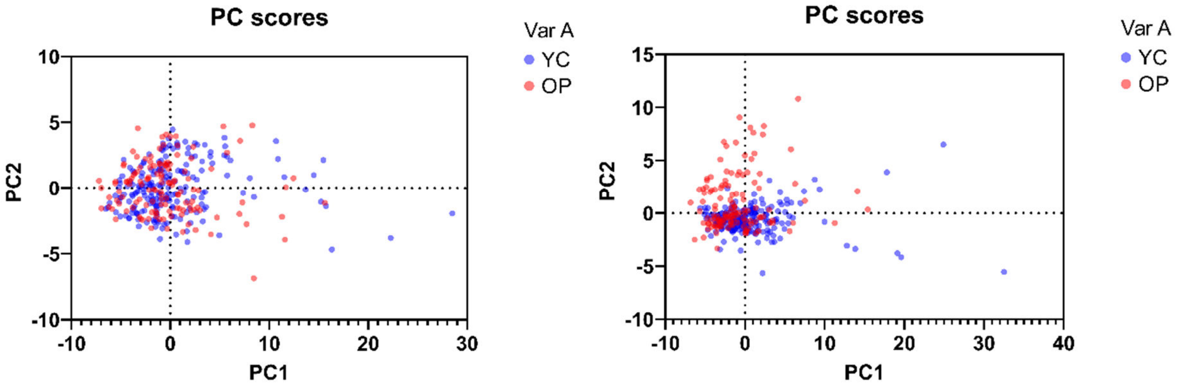

Supplementary Figure S4. (Left) PC Scores plot before introducing channel ratios. (Right) PC Scores plot after introducing channel ratios.
